# The Relationship between Acute and Chronic Pancreatitis with Pancreatic Adenocarcinoma: Review

**DOI:** 10.3390/diseases9040093

**Published:** 2021-12-20

**Authors:** Tamara Alhobayb, Rahul Peravali, Motaz Ashkar

**Affiliations:** 1 Department of Medicine, Division of Gastroenterology, School of Medicine, Washington University, St. Louis, MO 63110, USA; tamaraa@wustl.edu; 2 Department of Internal Medicine, School of Medicine, Washington University, St. Louis, MO 63110, USA; Peravali.r@wustl.edu

**Keywords:** acute pancreatitis, chronic pancreatitis, pancreatic cancer

## Abstract

Pancreatic ductal adenocarcinoma (PDAC) is a lethal disease with poor prognosis, leading to significant cancer-related mortality and an overall five-year survival rate of about nine percent. Acute and chronic pancreatitis have been associated with PDAC through common risk factors based on multiple epidemiological studies. Acute pancreatitis (AP) might be one of the earliest manifestations of PDAC, but evolving chronic pancreatitis (CP) following recurrent bouts of AP has been proposed as a risk factor for cancer development in the setting of persistent inflammation and ongoing exposure to carcinogens. This review aims to highlight the evidence supporting the relationship between acute and chronic pancreatitis with PDAC.

## 1. Introduction

Burden of pancreatic ductal adenocarcinoma (PDAC) is well-recognized globally, and it is estimated as the 11th most common cancer in the world based on accumulative data from 2018 [1]. PDAC incidence varies across the world, with higher rates in developed populations from North America (7.4 per 100,000 people) and Western Europe (7.3 per 100,000 people) [1,2]. PDAC is more common in men (4.9 per 100,000) than women (3.6 per 100,000) and increases in both sexes with age [1,2]. Importantly, PDAC annual incidence is increasing by 0.5–1.0% [3], and remains a lethal disease with poor prognosis, leading to significant cancer-related mortality with an overall five-year survival rate of about nine percent [4]. Rapid growth and lack of a reliable screening modality make diagnosing PDAC challenging, which results in disease discovery at advanced incurable stages [4]. By 2030, PDAC is projected to become the second leading cause of cancer-related mortality [3].

PDAC accounts for more than 90% of all pancreatic tumors [5]. Accumulating evidence suggests that pancreatitis is a risk factor for PDAC. Evolving chronic pancreatitis (CP) following recurrent bouts of acute pancreatitis (AP) has been proposed as risk factor for cancer development in the setting of persistent inflammation and ongoing exposure to carcinogens. Virchow’s observation of inflammatory cells within neoplastic tissue suggested correlations between inflammation and future dysplasia [6], proven by development of various cancers from sites of chronic irritation and inflammation, including tumors of the lung, GI tract, skin, and urinary bladder. This review aims to highlight the evidence supporting the relationship between acute and chronic pancreatitis with PDAC.

## 2. Association between Pancreatitis and Pancreatic Cancer

### 2.1. Acute Pancreatitis and Pancreatic Cancer

The association between pancreatitis and pancreatic cancer is well-established, and it is mostly driven by CP. Over the past few decades, there has been an ongoing debate on whether AP carries the same risk of developing pancreatic cancer, and multiple studies aimed to quantify such risk. Kirkegard et al. conducted a large nationwide matched cohort study in Denmark, which included 40,000 patients with AP. This work concluded that the risk of PDAC in patients with AP was two-fold higher compared to that in the general population over a 5–10-year follow-up period [7]. These observations were similar to the findings from US studies utilizing the VA system database [8,9]. Bansal et al. published a case-control study including about 2600 veterans with PDAC and noncancer controls. This work demonstrated higher likelihood of having a history of AP preceding PDAC (OR 1.76; 95% CI: 1.28–2.41) [8]. Another Taiwanese study assessed AP and PDAC association [10] by evaluating 747 hospitalized patients with AP and 5976 controls. This work observed significantly higher five-year risk of developing PDAC in the AP cohort compared to that in controls (HR 9.10; 95% CI: 3.81–21.76). A large population-based study in Sweden reproduced similar conclusions of elevated PDAC risk in patients with AP in the first few years, but interestingly, this decreased over time [11]. Two meta-analyses were conducted to ascertain the effect of AP and PDAC association. Zhang et al. meta-analysis of four cohort studies estimated a pooled relative risk of 8.30 (95% CI: 4.27–16.13) [12]. Another meta-analysis of 11 observational studies, suggested higher PDAC incidence after AP with estimated pooled relative risk of 7.81 (95% CI: 5.00–12.19) [13]. Although these observations highlighted a robust association between AP and PDAC, the question of whether this is a causal effect or if AP is a presenting symptom of PDAC remains unanswered.

### 2.2. Chronic Pancreatitis and Pancreatic Cancer

Multiple studies have shown that CP is highly associated with PDAC [8,14,15,16,17,18,19,20]. A multicenter, retrospective cohort study conducted by the International Pancreatitis Study Group showed that the cumulative risk of PDAC in patients with CP is 1.8% and 4% at 10 and 20 years, respectively, a rate which is from 15- to 16-fold greater than that of the general population [14]. Importantly, this risk was independent of sex, ethnicity, and type of pancreatitis (alcoholic and nonalcoholic). A single-center prospective cohort study verified these observations, concluding that CP patients have higher 5- and 10-year cumulative incidence of developing PDAC (1.1% and 1.7%) compared to that in age- and sex-matched controls [15]. Most convincingly, a meta-analysis of six cohort studies and one case-control study showed a pooled relative risk estimate of 13.3 for PDAC among patients with CP [16], which suggested that about five percent of patients with CP will develop PDAC within a 20-year follow-up period. These efforts proposed the higher causative association between long-standing CP and future PDAC development.

## 3. Pancreatitis and Pancreatic Cancer Risk Factors Overlap

The specific cause of PDAC remains elusive, but higher incidence has been observed in cohorts with certain environmental factors (Table 1). Cigarette smoking is highly associated with PDAC with a relative risk between 1.7 and 2.2 [21,22]. The International Agency for Research on Cancer has confirmed that cigarette smoking is causally linked with PDAC [23]. Studies have shown that risk of PDAC is nearly two times higher in smokers than in nonsmokers, and the risk increases with the duration of smoking and number of cigarettes smoked daily [24,25,26]. Diet is also a risk factor for PDAC. Red or processed meat is associated with increased incidence of PDAC; in contrast, nuts, vegetables, and fruits have been shown to be protective [27,28,29,30]. Obesity and diabetes mellitus are also linked to a higher PDAC risk [31,32,33]. In addition to environmental factors, individuals with inherited genetic disorders (e.g., Lynch syndrome, Li–Fraumeni syndrome, Peutz–Jeghers syndrome, familial adenomatous polyposis) or deleterious mutations in certain genes (e.g., K-ras, p53) carry a significantly increased risk for PDAC [34,35,36].

Detailed epidemiological studies have identified shared risk factors between pancreatitis and PDAC [16,42]. Given the overlapping risk factors, the relationship between pancreatitis and pancreatic cancer can be attributed to confounding. However, studies have shown that pancreatitis increases the risk of PDAC independently of risk factors. For example, one pooled analysis of ten case control studies (5048 cases of PDAC and 10,947 controls) showed a nearly three-fold association between previous pancreatitis and PDAC after controlling for potential confounders and effect-modifying covariates such as smoking history, alcohol intake, BMI, and history of diabetes mellitus [43]. The relationship between AP, CP, and PDAC is likely continuous, such that pancreatitis is an intermediate stage between normal pancreatic function and PDAC. Conceptually, pancreatic insults from smoking, alcohol abuse, and diabetes increase the risk for AP, and repeated bouts of AP increases the risk of progression to CP, which in effect can lead to carcinogenesis by fostering an environment that supports tumor formation.

## 4. Pathophysiology of Pancreatic Cancer in Pancreatitis

### 4.1. Acute Pancreatitis and Pancreatic Cancer Mechanisms

Many theories were proposed in order to explain the association between AP and PDAC. The main proposed mechanism is disease progression from acute to recurrent to chronic inflammation, which alters the pancreatic environment and facilitates dysplasia-to-neoplasia transition [42]. Another theory is that AP can cause genetic alterations that lead to accelerated formation of PDAC. This was suggested by Carrière et al. who observed that mice expressing oncogenic K-ras had rapid PDAC development after being subjected to two brief episodes of AP [44]. A different mechanistic theory is that AP is actually the first symptomatic presentation of PDAC secondary to tumor ductal obstruction, rather than being a predisposing risk factor [45]. In addition, most of the studies that found a strong association between AP and PDAC found a higher risk in the first two years after AP, after which the risk subsequently decreased. This suggests that AP is not a PDAC cause, but rather the cancer masquerading as inflammation.

### 4.2. Chronic Pancreatitis and Pancreatic Cancer Mechanisms

Carcinogenesis in CP is mediated by inflammatory processes. Chronic inflammation is characterized by sustained tissue damage, cellular proliferation, and tissue repair [46]. Uncontrolled cellular proliferation can lead to atypical cell production and carcinogenesis [47]. Diseases with chronic inflammation (e.g., Barrett’s esophagus, chronic gastritis, ulcerative colitis) have an increased risk of cancer of the affected organ [48]. Similarly, chronic pancreatic inflammation in CP can favor the malignant transformation of pancreatic ductal cells and lead to dysplasia and cancer. The specific cellular mechanisms underlying pathogenesis of pancreatic cancer in CP are far from clear, but research suggests interactions between inflammatory, proliferatory, and fibrotic pathways. Uncontrolled inflammation of the pancreas leads to activation and recruitment of cytokines and chemokines, which promote a microenvironmental milieu that favors progression from inflammation to malignancy [49]. Additionally, inflammation can enhance the activity of transcription factors, such as nuclear factor kappa B (NF-kB), which upregulate genes that promote carcinogenesis [50]. Inflammation also activates quiescent pancreatic stellate cells, which promote fibrosis and pancreatic injury [51]. Cytokines, transcription factors, and pancreatic stellate cells synergistically work together to promote oncogenesis in CP.

Chronic inflammation activates cytokines that induce localized tissue destruction and organ damage. Several proinflammatory cytokines, such as tumor necrosis factor-alpha (TNF-α), interleukin-6 (IL-6), interleukin-8 (IL-8), and interferon-y (IFN-y), are increased in both CP and PDAC [52,53]. Abnormal cytokine expression in CP can promote pancreatic carcinogenesis via a multitude of mechanisms. TNF-α, for example, is a potent cytokine that is released during the early stages of inflammation. In CP, TNF-α can upregulate the expression of platelet derived growth factor (PDGF), a growth factor that stimulates fibrogenesis [52]. In addition, TNF-α upregulates transforming growth factor-alpha (TGF-α), an oncogene that can increase cell proliferation and promote cancer [54]. Finally, TNF-α can inhibit the apoptosis of PDAC cells by activating NF-kB, an important transcription factor that has been implicated in many different cancers [55]. IL-6 is another important proinflammatory cytokine that is elevated in CP and PDAC. IL-6 can induce acinar cell damage in mice and has been shown to promote PDAC cell migration and invasion [56]. In one study, blockade of IL-6 resulted in reduced tumor progression in murine models of PDAC [57].

In addition to proinflammatory cytokines, upregulation of certain transcription factors can promote carcinogenesis in CP. NF-kB is upregulated in a significant number of human cancers, including pancreatic, colorectal, and lung cancers [58,59]. Under normal conditions, NF-kB is bound to inhibitory proteins (IkBs) that trap the transcription factor in the cytoplasm and keep it inactive. In chronic inflammatory conditions such as CP, the inhibitory proteins are degraded, and NF-kB translocates to the nucleus where it increases the transcription of proteins that promote oncogenesis [60]. Interestingly, NF-kB is associated with increased severity of CP and is constitutively activated in most PDAC cases [60,61]. NF-kB has been implicated in promoting cancer progression via a variety of mechanisms, including increasing cancer cell proliferation and antiapoptotic and inflammatory signals [62]. For example, studies have shown that NF-kB promotes cell cycle activity by activating cyclin D1, a protein that is upregulated in several PDAC cell lines [63,64]. In addition, NF-kB has been shown to induce IL-8, an inflammatory cytokine that is constitutively expressed at high levels in pancreatic cancer cells [53,65]. Chronic inflammation in CP activates transcription factors like NF-kB, which in effect induce inflammatory and oncogenic genes that promote cancer development.

Pancreatic stellate cells (PSCs), which are normally quiescent, regulate extracellular matrix production and pancreatic tissue architecture [66]. In response to cytokines and growth factors secreted by inflammatory cells, PSCs undergo functional changes to become myofibroblast-like cells that promote pancreatic injury and fibrosis [66,67,68]. Multiple in vitro and in vivo studies have proven the role of activated PSCs in fibrogenesis in CP [68]. Interestingly, agents that inhibit the activation of PSCs in vitro have shown promise in preventing the development of chronic pancreatitis [69,70,71]. Accumulating evidence suggests that PSCs may also promote the progression of pancreatic cancer [72]. In fact, activated PSCs play a pivotal role in pancreatic fibrogenesis and help create a desmoplastic reaction that favors oncogenesis [66]. In one in vitro study, PDAC cells induced the desmoplastic reaction by stimulating PSCs via soluble growth factors [68]. In addition, the study showed accelerated tumor growth in the presence of PSCs [68]. PSCs play an essential role in promoting fibrogenesis in CP and fostering the desmoplastic environment necessary for PDAC.

## 5. Pancreatitis as an Initial Symptom of Pancreatic Cancer

Over the past few decades, multiple studies have shown that AP might be one of the earliest manifestations of PDAC [73,74,75,76]. In fact, about one percent of AP cases are caused by PDAC [9,77]. The reported prevalence of AP prior to pancreatic cancer ranges between 1.4% and 6%, and most cases of PDAC-induced AP follow a mild disease course [75,78,79].

A large population-based study looking at both Danish and US cohorts found that pancreatic head tumors were more frequent in patients with AP. They also found that tumors identified in patients presenting with AP were early stages of PDAC and more amenable to resection, leading to better survival compared to those with no history of AP [78]. These results were similar in a study by Dzeletovic et al., which showed lower cancer-related mortality in patients with history of AP preceding their cancer diagnosis. In addition, survival was better compared to that of patients with no AP history (325 days vs. 387 days; *p* = 0.003) [80]. These observations were likely explained by earlier clinical presentations due to the painful nature of pancreatitis. Consequently, patients are diagnosed earlier, at a point where curative resections can be offered. Dzeletovic et al. concluded that 62% of patients with AP were able to undergo curative surgery compared to 43% in the non-pancreatitis group (*p* = 0.003). Comparative data from Lupinacci et al., which included PDAC patients who underwent a curative resection, found smaller tumor sizes (<4 cm) in patients presenting with AP compared to those in non-AP cases [81].

In contrast, Feng et al. investigated the survival rate in PDAC patients with moderate and severe AP compared to patients without AP. Their results showed a decrease in survival time in AP patients compared to that of patients with no prior pancreatitis [82]. However, this is explained by the impact of severe peripancreatic inflammation leading to difficult surgical resection with higher postoperative complications, hence poor survival [82,83]. A study by Li et al. investigated the optimal timing of surgical intervention in patients with AP with the goal of minimizing rates of postoperative complications. This work concluded that earlier surgery was significantly associated with postoperative complications, and the cutoff of 24.5 days was the optimal cutoff point for surgical resection (*p* = 0.025) [84]. However, the generalizability of this finding is limited by inclusion of patients with only a mild course of pancreatitis.

There are multiple predictors that were shown to be associated with PDAC in the setting of AP. Idiopathic pancreatitis was found to be a significant predictor in a large Danish cohort, with an adjusted hazard ratio of 2.52 (95% CI: 1.83–3.47) [7]. Other important risk factors that were described in the literature were age higher than 50 at diagnosis of AP, new-onset diabetes mellitus, and findings of CP at the time of AP diagnosis with alarm symptoms, including weight loss [77,80]. A Chinese study evaluating clinical predictors of underlying malignancy at the time of AP diagnosis found unexplained dilation of the main pancreatic to be highly suggestive of tumor presence (OR 417.83; 95% CI: 80.40–2171.42). Interestingly, mild AP course was found to be a predictor of PDAC, but in contrast, severe pancreatitis was not associated with new PDAC diagnosis [77,79]. Therefore, presence of PDAC predictors in patients with unexplained AP should elevate clinicians’ index of suspicion to aggressively evaluate and diagnose cancer early utilizing a combination of imaging and endoscopic modalities.

## 6. Discussion

PDAC remains a lethal disease with an extremely poor prognosis. According to the American Cancer Society 2019 data, approximately 56,770 people were diagnosed with and 45,750 died from PDAC in the United States. Unfortunately, PDAC disease incidence continues to grow by 0.5–1.0% annually, and by 2030, PDAC is projected to become the second leading cause of cancer-related mortality [3]. Accumulating and convincing evidence suggests that pancreatitis can be a sign of PDAC, but also might be a neoplastic precursor due to carcinogenesis either mediated by inflammatory processes or secondary to overlapping proinflammatory risk factors (e.g., smoking, alcohol use, diabetes mellitus, and obesity) that can further expedite cancer development [27]. It has been estimated that about 30% of PDAC could be prevented with the prevention of smoking [85]. Interestingly, the risk associated with smoking is reduced to the levels of a nonsmoker after 10 years of smoking cessation [85]. Moreover, dietary modification is important in preventing PDAC. Studies have shown that high consumption of red meat is associated with a greater risk of PDAC, whereas high fruit, vegetable, and nut intake is protective [29,86,87].

Therefore, the best preventive strategy against PDAC in patients with pancreatitis is risk reduction of modifiable risk factors.

Finally, it is important for clinicians to be able to risk-stratify and identify patients with PDAC earlier in their disease course in order to offer curative treatment. This requires special attention to older populations, patients with new diabetes mellitus diagnosis, smokers, and patients presenting with idiopathic pancreatitis. More studies are needed to establish guidelines and strategies in identifying these patients. 

## Figures and Tables

**Table 1:** PDAC risk factors.

Risk Factor	Increased PDAC Risk (RR)
BMI > 40	2.76 [32]
Type 2 diabetes mellitus	1.8–2.1 [37,38,39]
History of gallstones	1.7 [40]
Current cigarette use	1.7–2.2 [21,22]
>3 alcoholic drinks per day	1.22 [41]
Acute pancreatitis	7.81 [13]
Chronic pancreatitis	13.3 [16]
Hereditary pancreatitis (*PRSS1* mutation)	69 [16]

## Data Availability

Data sharing not applicable.
